# Comparative genomics profiling of clinical isolates of *Aeromonas salmonicida *using DNA microarrays

**DOI:** 10.1186/1471-2164-7-43

**Published:** 2006-03-07

**Authors:** John HE Nash, Wendy A Findlay, Christian C Luebbert, Oksana L Mykytczuk, Simon J Foote, Eduardo N Taboada, Catherine D Carrillo, Jessica M Boyd, Duncan J Colquhoun, Michael E Reith, Laura L Brown

**Affiliations:** 1 Institute for Biological Sciences, National Research Council of Canada, 100 Sussex Drive, Ottawa, Ontario, K1A 0R6, Canada; 2 Institute for Marine Biosciences, National Research Council of Canada, 1411 Oxford Street, Halifax, Nova Scotia, B3H 3Z1, Canada; 3 National Veterinary Institute, Department for Fish Health, Post Box 8156 Dep., 0033 Oslo, Norway

## Abstract

**Background:**

*Aeromonas salmonicida *has been isolated from numerous fish species and shows wide variation in virulence and pathogenicity. As part of a larger research program to identify virulence genes and candidates for vaccine development, a DNA microarray was constructed using a subset of 2024 genes from the draft genome sequence of *A. salmonicida *subsp. *salmonicida *strain A449. The microarray included genes encoding known virulence-associated factors in *A. salmonicida *and homologs of virulence genes of other pathogens. We used microarray-based comparative genomic hybridizations (M-CGH) to compare selected *A. salmonicida *sub-species and other *Aeromonas *species from different hosts and geographic locations.

**Results:**

Results showed variable carriage of virulence-associated genes and generally increased variation in gene content across sub-species and species boundaries. The greatest variation was observed among genes associated with plasmids and transposons. There was little correlation between geographic region and degree of variation for all isolates tested.

**Conclusion:**

We have used the M-CGH technique to identify subsets of conserved genes from amongst this set of A*. salmonicida *virulence genes for further investigation as potential vaccine candidates. Unlike other bacterial characterization methods that use a small number of gene or DNA-based functions, M-CGH examines thousands of genes and/or whole genomes and thus is a more comprehensive analytical tool for veterinary or even human health research.

## Background

*Aeromonas salmonicida*, the causative agent of furunculosis in salmonid fish, is a non-motile, Gram-negative bacterium, and one of the most studied bacterial pathogens of fish. Furunculosis is an important disease in wild and cultured stocks of fish with the potential for severe negative economic impact. The Canadian Aquaculture Industry Alliance estimated the total direct and indirect costs of infectious diseases within the Canadian aquaculture industry at over $400 M annually, with furunculosis contributing approximately $4 M annually to these losses [1]. *A. salmonicida *is not limited to salmonids and many species of fish are affected. Several excellent reviews of *A. salmonicida *and furunculosis are available [2-4].

Although Bergey's Manual of Systematic Bacteriology [5] recognizes five subspecies of *A. salmonicida*: *salmonicida*, *achromogenes*, *masoucida*, *smithia*, and *pectinolytica*, many laboratories currently classify *A. salmonicida *subsp. *salmonicida *as "typical" and any isolate deviating phenotypically as "atypical". Morphological and biochemical differences are used to distinguish typical and atypical isolates. These are pigment production, colony size and growth rate, haemolysis, and sucrose fermentation [4,6,7]. *A. salmonicida *subsp. *salmonicida *(i.e. typical) isolates grow well on blood agar with large colonies, produce brown, diffusible pigment, are haemolytic and do not ferment sucrose. Historically, typical strains are thought to be extremely homogenous [8,9], and therefore any deviation in any of these characteristics has been considered enough evidence to classify a strain as "atypical" [8,10]. However, some of these parameters are subjective, and are largely based on the experience of the operator/microbiologist, possibly leading to misclassification. Molecular techniques are not yet used on a regular basis in many laboratories that type *A. salmonicida*. Although typical isolates are generally phenotypically homogenous some variation does exist [10], particularly with regard to the production of hemolysin and degradation of casein. Antibiotic resistance patterns have shown potential as epizootiological markers in specific geographical origins. Typing schemes based on biochemical and other phenotypic methods are dependent on many factors, including plastic bacterial phenotypes and the often low consistency of inter-laboratory testing [11].

Genetic analysis provides a more stable basis for microbiological investigation than phenotypic methods. Several molecular techniques, including finger-printing by Randomly Amplified DNA Polymorphism (RAPD-PCR) [12,13], Amplified Fragment Length Polymorphism (AFLP) [14], Pulsed-Field Gel Electrophoresis (PFGE) [9,15], Restriction Enzyme Fragmentation Patterns (REFP) [16], plasmid profiling [17,18], and ribotyping [19,20], have been used to study different strains of A*. salmonicida*. While the results generally support the phenotypical evidence that typical strains are genetically homogenous and may well be clonal, and that atypical isolates are heterogeneous, there is little congruence between the techniques in establishing relationships between subspecies, strains and isolates. Austin *et al*. [1998] showed that among 52 isolates of atypical *A. salmonicida *taken from a wide host and geographical range, there was no agreement between PCR, RAPD, ribotyping, or phenotypic typing methods [8].

The use of genome arrays containing whole genomes or large sets of genes, either as a result of high-coverage genome sequencing or selected after suppressive subtractive hybridization have been used to study genome variability among strains of bacterial pathogens, including *Campylobacter jejuni *[21], *Flavobacterium psychrophilum *[22], *Listeria monocytogenes *[23], and *Burkholderia *species [24]. To examine the genomic diversity of virulence factors among clinical isolates of *A. salmonicida *and other aeromonads, we constructed a DNA microarray of 2024 selected virulence-related genes from *A. salmonicida *subsp*. salmonicida *strain A449. A449 is a wild-type typical virulent clinical isolate which has been sequenced and it is currently undergoing annotation.

We are undertaking a research program to examine the molecular mechanisms of interaction between *A. salmonicida *and Atlantic salmon (*Salmo salar *L.). The applied goal of this program is to develop vaccines, vaccine delivery systems and other health management tools. In order to develop vaccines we must first identify virulence-associated genes as potential vaccine candidates, the process known as "reverse vaccinology" [25]. Accurate typing is important in searching for vaccine candidates if there are known differences in pathogenicity of the sub-species, or if there is variation between the degree of conservation of virulence genes within each subspecies. Although all subspecies of *A. salmonicida *have been implicated in clinical cases in fishes, *Aeromonas salmonicida *subsp. *salmonicida *is more prevalent within salmonids and "typical" isolates are more often associated with disease outbreaks in salmon [3]. Moreover, there are indications that there are differences in the susceptibility and immune response of salmonids and other fishes to "atypical" isolates [14,26]. Therefore it is essential that any characterization or typing schemes be accurate, if health management strategies are to be based, even partly, on those schemes.

We used M-CGH to study the genetic relationships between *Aeromonas *species, subspecies and strains based on gene conservation profiles and to examine genomic diversity. We compared strains and isolates from selected geographic areas and host species to explore correlations between geographic or host origin and conservation or diversity of genes. These strains were selected to provide a diverse sample of clinical and laboratory isolates from the differing geographic regions and host species infected. Our goal was to identify a set of virulence-associated genes conserved across these strains to consider for future consideration as vaccine candidates. We hypothesized greater variability of these virulence genes between subspecies and no correlation between the "typical" and "atypical" classifications and gene variability. We also hypothesized positive correlation between the degree of variability and the functional category of the variable genes involved.

## Results

Comparative genomic hybridization (M-CGH) profiles for each of the *Aeromonas *isolates listed in Table 1 were obtained by competitively hybridizing labeled genomic DNA (gDNA) from the relevant isolate and from the A449 control strain to an *A. salmonicida *microarray comprising 2024 putative virulence genes, selected from a draft genome sequence of the wild type strain *A. salmonicida *subsp. *salmonicida *A449.

**Figure 1:**
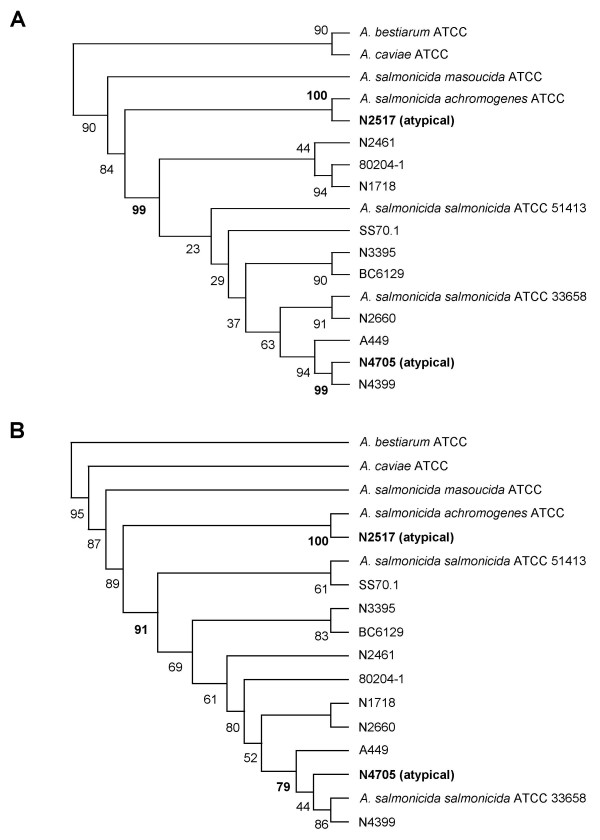
**Hierarchical clustering of *Aeromonas *strains based on M-CGH data for all genes on the microarray**. The dendrogram was produced using the MEV software from TIGR with Euclidean distance and average linkage clustering (n = 1,000 bootstrap iterations). Isolates in bold are atypical isolates that cluster with other known subspecies. The bootstrap values which lead to their cluster assignment are also in bold. All ATCC type strains are denoted "ATCC", and unless otherwise noted, all other isolates are *A. salmonicida *subsp. *salmonicida*. (A) Sample clustering based on all genes on the AsalChip1 microarray. (B) Sample clustering based on genes not assigned to the plasmid or transposon functional categories (i.e. "chromosomal" genes).

Hierarchical clustering of the seventeen *Aeromonas *isolates based on the overall variability in the M-CGH data is represented by the dendrogram in Figure 1A. As expected, the ten *A. salmonicida *subsp. *salmonicida *strains and the control strain A449 clustered together. One atypical strain (N4705) also clustered in this group. There is very strong support (99%) for the clustering of the eleven *A. salmonicida *subsp. *salmonicida *strains with the atypical strain N4705, but much lower bootstrap values for most branches within this main cluster. Figure 1B shows hierarchical clustering of the seventeen *Aeromonas *samples based only on "chromosomal" genes, after genes assigned to the plasmid and transposon functional categories were removed. The ten *A. salmonicida *subsp. *salmonicida *strains cluster with A449 and the atypical strain N4705. In each dendrogram, as expected, *A. bestiarum *and *A. caviae *showed the most divergence from the main cluster, followed by *A. salmonicida *subsp. *masoucida *and *A. salmonicida *subsp. *achromogenes*, consistent with the species/subspecies boundaries. It is interesting to note that one of the *A. salmonicida *atypical samples (N2517) clustered with *A. salmonicida *subsp. *achromogenes*, whereas the second atypical strain (N4705) clustered with the *A. salmonicida *subsp. *salmonicida *A449 control strain. These results suggest that neither of these should be considered *A. salmonicida *atypical strains.

**Figure 2:**
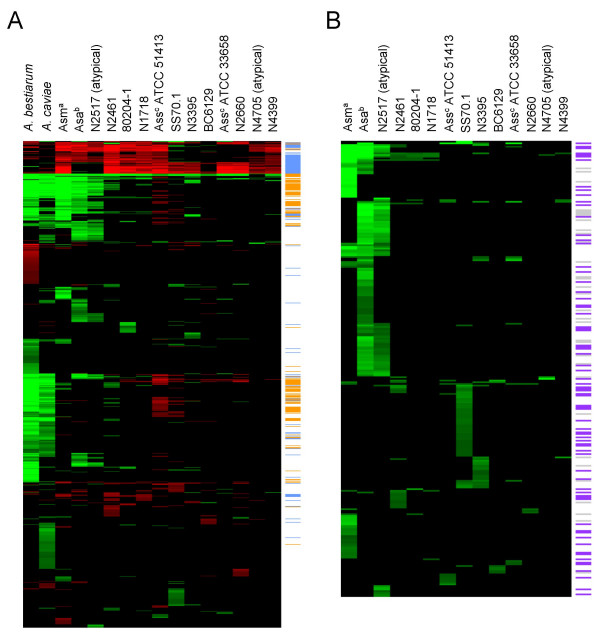
**Variable genes in *Aeromonas *samples**. Genes which were conserved (-1<Log Ratio <1) in all samples are removed. Red indicates genes with higher signal intensity for the tester than the control strain (higher copy number than strain A449). Green indicates genes with lower signal intensity for the tester than the control (divergent in sequence or missing or lower copy number). Gene order reflects results of hierarchical clustering of genes performed as described in Materials and Methods. The strains are ordered as in Figure 1, and unless otherwise noted, all other isolates are *A. salmonicida *subsp. *salmonicida*. ^a^Asm – *A. salmonicida *subsp. *masoucida*, ^b^Asa – *A. salmonicida *subsp. *achromogenes*, ^c^Ass – *A. salmonicida *subsp. *salmonicida*. (A) All genes which are divergent in at least one of the sixteen *Aeromonas *isolates. Blue bars indicate genes associated with the plasmid category and orange bars indicate genes associated with the transposon category as described in Material and Methods. (B) Subset of genes which are divergent (Log Ratio< -1) in the fourteen *A. salmonicida *strains and were not assigned to the plasmid or transposon functional categories. Genes predicted to code for OMPs (purple) and for flagella/pili proteins (grey) are indicated.

The M-CGH data for the sixteen *Aeromonas *samples ordered with hierarchical clustering of the genes is shown in Figure 2. Genes were considered to be conserved if the signal differed by less than a factor of two between the tester and the control (Log Ratios of between -1.0 and +1.0), as described by Taboada *et al*., 2004 [21]. The lower cutoff of -1.0 is fairly conservative as we have shown previously that it encompasses over 99.5% of highly conserved genes but less than 1% of missing genes [27]. To highlight the variable genes, the Log Ratio value was set to 0 (black) for conserved genes, and genes which were conserved in all of the samples have been removed. Genes with Log Ratio values less than -1.0 (green) were considered to be divergent in sequence or absent from the tester strain, while those with Log Ratios values greater than +1.0 (red) were expected to be present in higher copy number in the tester than in the control strain. As shown in Figure 2A, clusters with the highest variability across the samples correspond to plasmid- and transposon-associated genes. As expected, *A. bestiarum *and *A. caviae *have higher numbers of variable genes than the *A. salmonicida *strains. Figure 2B shows the subset of genes that are not associated with transposons or plasmids but are divergent in at least one of the fourteen *A. salmonicida *samples. These divergent "chromosomal" genes show some clustering of genes coding for outer membrane proteins and flagella/pili.

The number of genes divergent or absent according to the function categories assigned in Table 2 and by species/strain is presented in Table 3. The highest degree of divergence compared to A449 can be seen for *A. caviae *and *A. bestiarum*, followed by the type strains of *A. salmonicida *subspp. *masoucida *and *achromogenes *as well as one of the atypical samples (N2517). There was much less variation between the *A. salmonicida *subsp. *salmonicida *strains and the other *A. salmonicida *atypical strain (N4705). Of the *A. salmonicida *subsp. *salmonicida *strains, N2461, isolated from turbot, and SS70.1, the only isolate from a Pacific salmon species (coho salmon), showed the highest degree of variability among chromosomal genes. These data notwithstanding, there does not appear to be a strong correlation between the level of genomic variability and either host or geographical origin.

**Figure 3:**
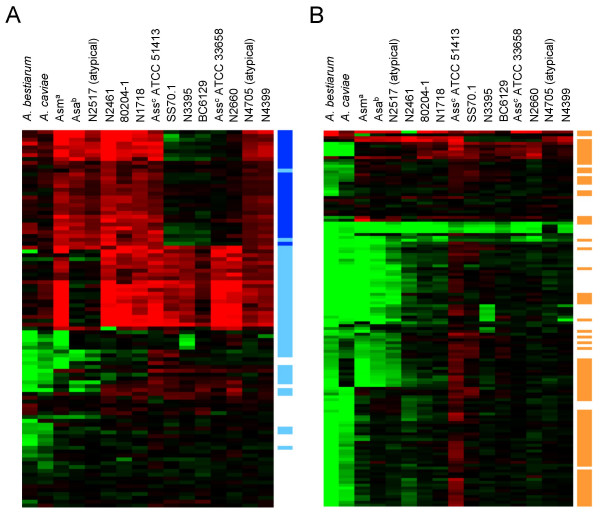
**Variability of genes associated with plasmids and transposons in 16 *Aeromonas *strains and species**. Red indicates genes with higher signal intensity for the tester than the control strain (higher copy number than in strain A449). Green indicates genes with lower signal intensity for the tester than the control (divergent in sequence or missing or at lower copy number). Gene order reflects results of hierarchical clustering of genes performed as described in Materials and Methods. The strains are ordered as in Figure 1, and unless otherwise noted, all other isolates are *A. salmonicida *subsp. *salmonicida*. ^a^Asm – *A. salmonicida *subsp. *masoucida*, ^b^Asa – *A. salmonicida *subsp. *achromogenes*, ^c^Ass – *A. salmonicida *subsp. *salmonicida*. (A) All genes from the plasmid functional category. The blue bars (both light and dark) indicate genes found on plasmid 5, and the dark blue bars correspond to predicted TTSS genes. (B) All genes from the transposon functional category. The orange bars indicate genes with strong sequence similarity to known transposases.

The M-CGH data for genes associated with mobile elements (plasmids and transposons) in the sixteen *Aeromonas *samples is shown in Figure 3. Hierarchical clustering of the plasmid-associated genes (Figure 3A) shows clustering of highly variable genes matching a known plasmid of ~155 kb [plasmid 5, M. Reith – unpublished data]. A subset of genes on this plasmid matches type three secretion system (TTSS) genes found in other bacteria, which is unsurprising considering that TTSS genes can be plasmid-borne in *A. salmonicida *subsp. *salmonicida *[28]. We observed genomic variability consistent with the lower intensity signal for some plasmid genes, including TTSS genes, for several strains (Figure 3A). Of these, the strain SS70.1 is an avirulent, laboratory-derived strain created by treating an A. *salmonicida *subsp. *salmonicida *strain with ethidium bromide [29,30]. This likely resulted in the loss of at least one plasmid [data not shown], which would contribute to this observation. On the other hand, many genes on plasmid 5 appear to be present in higher copy number in many of the tester strains than in A449. Figure 3B shows the M-CGH data for genes associated with transposons. Many of these genes appear to be highly divergent, present at lower copy number, or missing in various tester strains, and many of the genes match known transposases.

**Figure 4:**
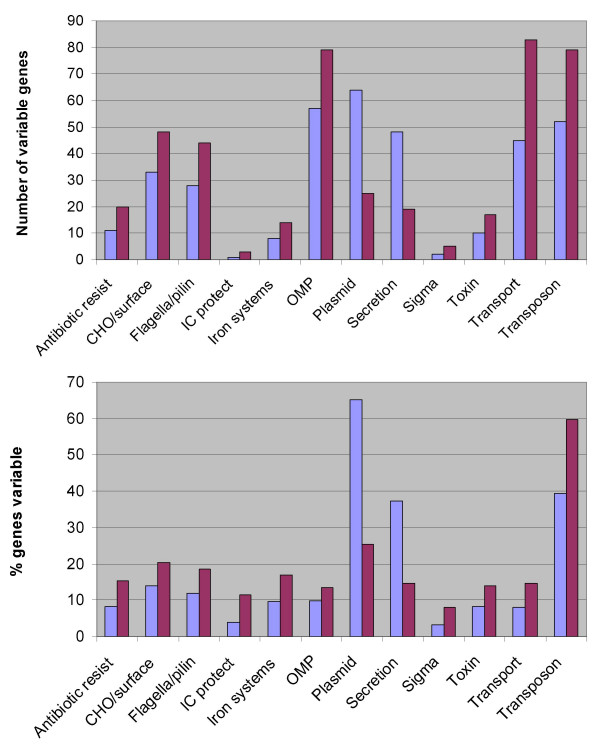
**Variability of genes associated with different functional categories**. Blue bars correspond to genes which were present at higher copy number (Log Ratio >1) in at least one of the *A. salmonicida *isolates that in A449. Red bars correspond to genes which were divergent or missing (Log Ratio <-1) in at least one of the *A. salmonicida *isolates. Upper panel: Number of variable genes assigned to each functional category. Lower panel: Percent of genes in each functional category which were variable.

The number of variable genes assigned to each functional category is shown in Figure 4, as well as the percent of genes in each category that were observed to be variable. Within the function categories other than transposon or plasmid, most genes that appeared to be divergent or missing in various *Aeromonas *strains are associated with transport, surface carbohydrate biosynthesis, outer membrane, and flagella/pili proteins. In contrast, genes associated with secretion were most likely to be present in higher copy numbers in the tester strains compared to A449. In summary, the highest degree of variability was seen in *Aeromonas *genes associated with mobile elements: the plasmids or the transposons, whereas genes within the chromosome of the bacterium displayed the lowest level of variability.

## Discussion

In this study, patterns of variability of the 2024 selected virulence-associated genes of *A. salmonicida *subsp. *salmonicida *strain A449 within the sixteen various test isolates of *A. salmonicida *and other *Aeromonas *species were examined. It is noteworthy that genes divergent and absent in some tester strains seem to be distributed across the entire A449 genome sequence and across all the predicted functional categories whereas genes shown to be at higher copy number than in the reference strain were clustered in selected loci associated with plasmids. The number of divergent and absent genes correlated strongly with species and subspecies boundaries. For *A. bestiarum *and *A. caviae *~16% of the genes were divergent or missing, whereas for the *achromogenes *and *masoucida *subspecies ~8% of the genes were divergent or missing. The range across the various *A. salmonicida *subsp. *salmonicida *strains was between 0.3% and 2%, again reinforcing the single clone theory for this species. Overall, for the 14 *A. salmonicida *isolates studied, approximately 19% of the genes were divergent in at least one sample. For comparison, our previous meta-analysis of M-CGH studies of 97 strains of *C. jejuni *showed ~33% of the genes in the genome were divergent in at least one strain [21]. It is likely that as we examine more strains of *A. salmonicida *the number of divergent genes will increase. One interesting finding is that almost 70% of the virulence-associated genes on the DNA microarray are conserved across all the *Aeromonas *species. Because more than 80% of these highly conserved genes appear to be chromosomal, this suggests that the divergence of *A. bestiarum*, *A. caviae*, and *A. salmonicida *may have occurred fairly recently.

The majority of *A. salmonicida *subsp. *salmonicida *isolates studied were isolated from Atlantic salmon and gave homogenous results, i.e., there is no correlation between host or geographic origin and M-CGH patterns in subsp. *salmonicida *isolates. Highest variability amongst the subsp. *salmonicida *isolates was observed in an isolate (SS70.1) from coho salmon and in an isolate (N2461) from turbot. However, more isolates from Pacific salmon should be analyzed before drawing definite conclusions on the variation between isolates from Atlantic and Pacific salmon. The relatively low number of divergent genes in most of the *A. salmonicida *subsp. *salmonicida *strains supports the findings of Garcia *et al*. who identified a single clone of *A. salmonicida *subsp. *salmonicida *as responsible for most outbreaks of disease worldwide [9].

Differentiation of typical and atypical strains of *A. salmonicida *is important economically if it can be shown that there are significant differences in the degree of variation in the genes associated with virulence and pathogenicity, and which affect the immune response of the host. If this is the case, then accurate typing methods and differentiation between typical and atypical strains and subspecies are required for diagnostic methods and for vaccine development. Lund *et al*. have shown variation in the protective ability of some commercial furunculosis vaccines (whole bacterins made with typical strains) against atypical strains infecting farmed spotted wolffish [14] and halibut (cited in [14]). In this context, M-CGH provides an objective means to type clinical isolates based upon the presence, absence or divergence of a large number of genes.

Phenotypical variation amongst subsp. *salmonicida *isolates is rare but has been described [31]. The increased resolution of M-CGH compared to phenotypic typing can be illustrated on consideration of strain N4705 which was considered an atypical strain on phenotypical grounds. However, both M-CGH and subsequent pulsed-field gel electrophoresis [Colquhoun, unpublished results] suggest that this strain is very closely related to, and should perhaps more accurately be considered a typical strain. Similarly, atypical strain N2517, may be more accurately placed within the subspecies *achromogenes *based upon M-CGH results.

Further comparative studies will be required to establish the level of strain resolution capable by M-CGH for aeromonads. However, in the absence of complete genome sequence data for each *Aeromonas *strain used, M-CGH, which in this study uses over 2,000 separate genetic markers, represents a comprehensive, high- resolution methodology for comparing genome information. Recent work by our group used M-CGH to examine the quantitative relationship between the Log Ratio and probe/target identity, and these analytical processes were used in the study [27].

It is preferable that vaccine candidates be strongly conserved among strains that cause disease. The M-CGH data from these experiments will provide a list of conserved genes, sorted by function category, which can be further analyzed to generate these candidates. For example, there are bioinformatics programs available to predict sub-cellular location [32] to select candidates which may be exposed to the host immune system, or to predict adhesins [33]. These downstream analyses, in combination with M-CGH analysis will reduce the number of genes required to be cloned in order to test the immune response of their encoded proteins. These experiments are currently underway.

It must be noted that M-CGH does not reveal information about gene expression or the specific role of targeted genes in pathogenicity or host immune responses. However, it is a powerful tool in reverse vaccinology and M-CGH is a sensitive and comprehensive technique that can determine genomic variation between pathogen strains, and it also can be used as a screening tool for target identification, and/or a typing method.

## Conclusion

M-CGH is a powerful first screen for vaccine target identification, and is the basis of reverse vaccinology [25], as it facilitates identification of conserved and duplicated genes associated with virulent strains. This technique may also prove useful in strain typing and epidemiological studies.

Comparative analysis of genomic data of *A. salmonicida *and related organisms reveals candidates common to all clinical isolates or those strains shown to be associated with virulence and disease. Our work on knock-out mutants of A449 virulence genes, combined with proteomic analysis of selected isolates confirm the results of our M-CGH analyses and will enable us to select specific antigens for vaccine development [work in progress].

The findings reported here support our initial hypotheses that variability exists between subspecies, that there is no correlation between 'typical' and 'atypical' classifications and gene variability. We also found that there is a positive correlation between the degree of variability and the functional category of the genes involved.

Future work will include the use of microarray transcript profiling experiments to further focus the choice of target genes. This will allow us to develop an ongoing, dynamic list of vaccine candidates to be used in live challenges for virulence and protection assays and then in further vaccine development.

## Methods

### Bacterial strains

Isolates of *Aeromonas *spp. used in this study are presented in Table 1. The isolates were characterized as belonging to either typical or atypical groups using a limited number of biochemical tests and phenotypical characteristics. Important characteristics for differentiation were growth rates, colony size, production of brown diffusible pigment, hemolysin production, and to a lesser extent, production of acid from sucrose. The reference strain, A449 is a wild-type isolate of *A. salmonicida *subsp. *salmonicida*, kindly donated by Dr. William Kay, University of Victoria, British Columbia. All strains were grown on Tryptic Soy Agar (TSA, Difco) at 18°C for 24–48 hours before genomic DNA isolation.

### Selection of genes for DNA microarray construction

The draft genome sequence of *A. salmonicida *strain A449 [available upon request from the National Research Council of Canada, Institute for Marine Biosciences] was used as a source of the genes used in this study. ORFs were identified using the Glimmer software package [34], and used to search for homologs among the bacterial gene subset of Genbank [35] using the BLASTP program [36]. We selected 2024 ORFs for inclusion on a DNA microarray based upon one or more of the following criteria:

i) Similarity to known virulence factors from other bacteria based on visual examination of the BLASTP analysis of the *A. salmonicida *genome.

ii) Presence of "virulence-associated keywords" within the gene annotations of homologs returned from BLASTP analysis. These keywords and their associated functional categories are described in Table 2.

iii) Similarity to genes in the National University of Singapore Fish Pathogen Database [37].

DNA sequences corresponding to plasmids pAsa1, pAsa2 and pAsa3 were excluded from our microarray because they only had genes for self-existence, and no virulence factors [38]. Sequences corresponding to two then-newly-discovered plasmids (plasmids 4–5), which had not been fully characterized at the time the draft sequence was obtained, are included in the DNA microarray.

### Construction of an *Aeromonas salmonicida *A449 amplicon-based DNA microarray

PCR primers were designed successfully for each of the 2024 ORFs described above using the Primer3 program [39] controlled by an automated script as described previously [21]. Primer-selection parameters were standardized and included a similar predicted melting temperature (62 ± 3°C), uniform length (21 nt), and a minimum amplicon size of 160 bp. The average amplicon size was 890 bp (range 164 to 4268 bp). Generation of PCR amplicons and fabrication of DNA microarrays were as described [21]. Details on the construction of this microarray (AsalChip1) are available [40].

### Genomic DNA labelling

Genomic DNA was isolated as described [21], and fragmented by nebulization [41]. 100 μg of DNA in nebulization buffer [10 mM Tris; 1 mM EDTA (pH 8.0), 35% glycerol (v/v)] was placed in the chamber of an AeroMist Nebulizer (IPI Medical Products, Chicago, IL), and sheared by passing nitrogen gas through the chamber at 15 psi for 1 minute. The DNA was precipitated with ethanol and suspended in 100 μl of 10 mM Tris; 1 mM EDTA (pH 8.0). Typically, the DNA was fragmented to a range of 0.4 to 12 kb in size. 5 μg of fragmented gDNA were fluorescently labelled using direct chemical coupling with the Label-IT (Mirus Corp., Madison, WI) cyanine dyes Cy3 and Cy5 as recommended by the manufacturer. Probes were purified from unincorporated dyes by sequentially passing samples through SigmaSpin (Sigma, Oakville, ON) and Qiaquick (Qiagen, Mississauga, ON) columns. Labelled DNA sample yields and dye incorporation efficiencies were calculated using the Nanodrop ND-1000 spectrophotometer (Nanodrop, Rockland, DE).

### Microarray hybridizations

The hybridization profile for each strain was obtained by co-hybridizing labelled DNA from the tester strain and from the *A. salmonicida *A449 (control) strain to our microarray. DNA from tester strains was labelled with Cy5 and the control strain with Cy3. Dye swaps were performed on selected strains to test for potential dye-incorporation bias. Labelled samples were normalized by selecting tester/control sample pairs with similar dye incorporation efficiencies. Equivalent amounts (1 to 2 μg) of labelled tester and control samples were pooled, lyophilized, and then re-suspended in 35 μl of hybridization buffer [1 × DIGEasy hybridization solution (Roche, Laval, QC); 0.5 μg/μl of torulla yeast tRNA]. Probes were denatured at 65°C for 5 minutes and applied to the microarray. Hybridizations were performed overnight at 37°C under 24 × 42-mm glass cover slips in a high-humidity chamber. Microarrays were washed 2 × 5 minutes at 50°C in 1 × SSC with 0.1% SDS, then 2 × 5 minutes at 50°C in 0.5 × SSC, and 1 × 5 minutes at 50°C in 0.1 × SSC. Slides were spun dry (500 × g, 5 minutes) and stored in lightproof containers until scanned.

### Data acquisition and analysis

**Figure 5:**
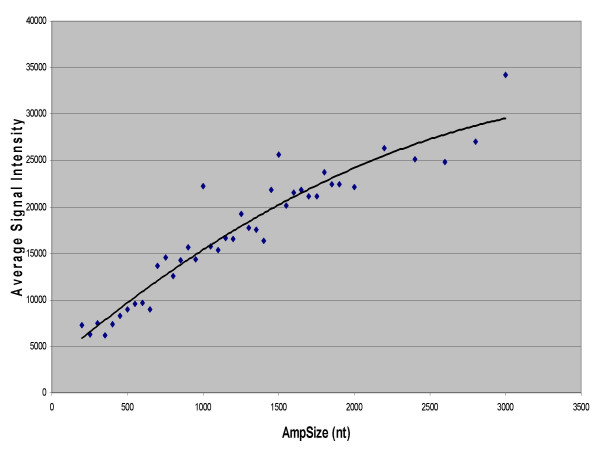
**Variation in hybridization signal intensity with amplicon length**. The average signal intensity in the control channel is plotted versus amplicon size. The amplicons were "binned" in 50 bp increments (to 1900 bp) then in 200 bp increments (from 2000 to 3000 bp). There were only 4 amplicons of size over 3000 bp.

Microarrays were scanned using a Chipreader laser scanner (BioRad, Mississauga, ON) according to the manufacturer's recommendations. Spot quantification, signal normalization and data visualization were performed using the program ArrayPro Analyzer (version 4.5; Media Cybernetics, Silver Spring, MD). Net signal intensities were obtained by performing local-ring background subtraction. "Tester signal" is defined as the signal intensity of the selected *Aeromonas *isolates labeled with appropriate fluorescent dye, while "control signal" is defined as the signal intensity of *A. salmonicida *strain A449 labelled with its appropriate fluorescent dye. The control signal increases with increasing amplicon size as shown in Figure 5. The ratio of tester signal to control signal for each gene was transformed to its base 2 logarithm [42], log_2 _[Tester Signal/Control Signal], referred to as "Log Ratio". Data from each channel were adjusted using cross-channel Loess normalization of the Log Ratio data and low intensity and anomalous spots were flagged and removed. Data was stored and archived using the BASE BioArray Software Environment [43].

Technical variation in our methodology was tested by selecting a subset of strains for replicate hybridizations, and treating the data from replicates separately throughout the various analyses. Consistency in the data was assessed by direct comparison of the lists of variable genes obtained from each replicate. Microarray data from sets of hybridizations were exported from BASE after removal of flagged spots, Loess normalization and averaging of data from duplicate spots. Filtering of genes based on the functional categories described in Table 2 was applied as required, and the results were analyzed using the MEV software package from TIGR [44,45]. Visualization and hierarchical clustering of microarray data, using Euclidean Distance metrics and Average Linkage Clustering, was performed in MEV using algorithms developed by Eisen *et al*. [46]. We generated sample trees as well as support trees based on bootstrapping genes with 1000 iterations to examine the variation of CGH profiles between the different isolates.

### Identification of plasmid and TTSS genes

Chromosomal and plasmid genes present on the array had not been distinguished from each other in the original construction phase. When the sequences of plasmids 4–5 became available, i.e. were separated from the chromosomal genome assembly as it progressed, the BLAST software package [47] was used to compare the nucleotide sequences of all ORFs on the *A. salmonicida *microarray chip to the sequences of plasmids 4–5, and thus identify them. We also created a database of all known bacterial TTSS genes and used BLAST to identify ORFs within the *A. salmonicida *genome sequence with high similarity to these genes. These results were used to generate gene lists for uploading into MEV to characterize observed gene clusters.

## List of abbreviations

M-CGH: microarray-based comparative genomic hybridization; gDNA: genomic DNA; ORF: open reading frame; nt: nucleotides; psi: pounds per square inch

## Authors' contributions

JHEN co-led the conception and design of the study, contributed to the design of the microarray, participated in the interpretation of data, drafted and co-wrote the manuscript. WAF carried out analysis and interpretation of microarray data, produced the figures, assisted in production of the microarray, and contributed to writing the manuscript. CCL and OLM performed microarray hybridizations, generated microarray data, and assisted in production of the microarray. SJF identified the genes and performed the annotation of the draft version of genome. ENT participated in the design of the study and interpretation of data. CDC participated in the design of the study and performed microarray hybridizations. JMB participated in the selection of virulence targets for the array, and contributed to writing the manuscript. DJC provided several strains, participated in the interpretation of data, and in the writing of the manuscript. MER provided the draft sequence of the genome, and contributed to writing the manuscript. LLB co-led the conception and design of study, designed and built the DNA microarray, participated in the interpretation of data, drafted and co-wrote the manuscript. All authors submitted comments on drafts, and read and approved the final manuscript.

## Figures and Tables

**Table 1:** *Aeromonas *strains analyzed by M-CGH in this study

**Strain**	**Species**	**Host species**	**Origin**	**Source^1^**
A449	*A. salmonicida salmonicida*	*Salmo trutta *(brown trout)	France	CC^4^
ATCC 51108	*A. bestiarum*	diseased fish	Scotland	CC^5^
ATCC 15468	*A. caviae*	guinea pig	France	CC^5^
ATCC 33659	*A. salmonicida achromogenes*	*S. trutta*	USA	CC^5^
N2517	*A. salmonicida *atypical	*Hippoglossus hippoglossus *(Atlantic halibut)	Norway	CC^6^
N4705	*A. salmonicida *atypical	*Scophthalmus maximus *(Turbot)	Norway	CC^6^
ATCC 27013	*A. salmonicida masoucida*	*Oncorhynchus masoucida *(Masou salmon)	Japan	CC^7^
ATCC 33658	*A. salmonicida salmonicida*	*Salmo salar *(Atlantic salmon)	France	CC^7^
ATCC 51413	*A. salmonicida salmonicida*	*S. trutta*	Finland	CC^7^
80204-1	*A. salmonicida salmonicida*	*S. salar*	NB^2^	LD^7^
BC6129	*A. salmonicida salmonicida*	*S. salar*	BC^3^	CC^8^
N1718	*A. salmonicida salmonicida*	*S. salar*	Norway	CC^6^
N2461	*A. salmonicida salmonicida*	*S. maximus*	Norway	CC^6^
N2660	*A. salmonicida salmonicida*	*S. salar*	Norway	CC^6^
N3395	*A. salmonicida salmonicida*	*S. salar*	Norway	CC^4^
N4399	*A. salmonicida salmonicida*	*S. salar*	Norway	CC^6^
SS70.1	*A. salmonicida salmonicida*	*Oncorhynchus kisutch *(Coho salmon)	NB^2^	LD^7 ^[30]

^1 ^Clinical Case (CC) or Lab-Derived (LD)

^2 ^New Brunswick, Canada (NB)

^3 ^British Columbia, Canada (BC)

^4 ^Dr. William Kay, University of Victoria, BC;

^5 ^American Type Culture Collection (ATCC)

^6 ^Norwegian Veterinary Institute, Oslo, Norway

^7 ^Dr. Gilles Olivier, Department of Fisheries and Oceans, Moncton, New Brunswick

^8 ^Dr. Joanne Constantine, BC Ministry of Agriculture, Food and Fisheries, Victoria, British Columbia

**Table 2:** Keywords from gene annotations used to identify virulence and virulence-associated genes, and assign functional categories

**Keyword**^a^	**Functional category**	**Relevance**
Aerolysin	Toxin	known virulence protein
antibiotic	Antibiotic resistance	antibiotic resistance/susceptibility
capsule	CHO/Surface factors	capsule biosynthesis/transport
catalase	Flagella/pilin; OMP	catalase/peroxidases
chemotax	IC protect; Toxin	chemotaxis proteins
chitin	Other; Toxin	chitinase
cpa	Flagella/pilin	pilus assembly complex
dismutase	IC protect	superoxide dismutase
extracel	Other	extracellular proteins
ferr	Iron systems; Transport;	ferredoxins, ferric transport
fimb	Flagella/pilin	fimbrial proteins
flag	Flagella/pilin	flagellar proteins
glycosyl	CHO/Surface factors	glycosyl transferases
hemolysin	Toxin	hemolysin/aerolysin
invas	OMP	invasions or invasion/adhesion
iron	Iron systems; Transport	iron transport/binding
Laf	Flagella/pilin	*Vibrio parahaemolyticus *chemotaxis protein
Lfg	Flagella/pilin	*V. parahaemolyticus *flagellar hook protein
lip	CHO/Surface factors	lipid/lipoprotein
macrophage	IC protect	macrophage toxins
membrane	OMP	outer/inner membrane proteins
OMP	OMP	outer membrane proteins
penicillin	Antibiotic resistance	penicillin resistance
peroxid	IC protect ; Toxin	peroxidase
pil	Flagella/pilin	pilin/pilus assembly protein
plasmid	Plasmid	plasmid-encoded
polymer	Sigma	DNA polymerase/Sigma factor
porin	OMP	outer membrane porin protein
protease	Other; Toxin	proteases
pseudopilin	Secretion	part of Type II secretion system
pul	Flagella/pilin	part of Type II secretion system
quorum	Other	quorum sensing proteins
receptor	OMP	membrane receptor proteins
resistance	Antibiotic resistance	antibiotic resistance
RTX	Toxin	RTX toxin (hemolysin)
secret	Secretion	secretion systems/secretory proteins
sial	CHO/Surface factors; Other;	sialic acid transport/synthetase
siderophore	OMP	siderophore receptor
sigma	Sigma	sigma factor
SOD	IC protect	superoxide dismutase
superoxide	IC protect	superoxide dismutase
Tad	Flagella/pilin	*Vibrio spp*. pilus assembly protein
tap	Other; Transport	ABC transporter/ATP-binding protein
toxin	Toxin	toxin genes
transferase	CHO/Surface factors; Other	glycosyl and other transferases
transport	Transport	transport proteins
transpos	Transposon	transposases
usher	Flagella/pilin	chaperone/usher type fimbriae
Yop	Secretion	Type III Secretion system gene in *Yersinia spp*.

^a^or portions of keywords selected to include grammatical variants, e.g. *invas *would select *invas*ion or *invas*ins, etc.

**Table 3:** Number of divergent genes (Log Ratio <-1) in each strain for each functional category

					*A. salmonicida *subsp. *salmonicida*										*A. salmonicida *atypical	
					
**Functional category:**	Abe^a^	Aca^b^	Asa^c^	Asm^d^	33658	51413	80204-1	BC6129	N1718	N2461	N2660	N3395	N4399	SS70.1	N2517	N4705
Antibiotic resist	11	14	7	5	0	0	0	0	0	0	0	4	0	6	3	0
CHO/surface	30	36	20	18	4	3	3	4	3	6	3	7	3	9	14	0
Flagella/pilin	50	30	29	12	1	1	0	0	0	1	1	1	1	2	16	0
IC protect	3	3	1	1	0	0	0	0	0	0	0	0	0	0	0	0
Iron systems	16	15	7	6	0	0	0	0	0	1	0	2	0	2	5	0
OMP	53	56	33	21	6	4	5	1	3	8	1	4	1	16	22	2
Other	24	43	22	13	4	3	3	3	3	4	3	3	3	7	18	0
Plasmid	28	25	8	10	0	0	0	0	3	4	0	5	1	4	6	0
Secretion	22	17	4	7	1	0	0	1	0	1	0	4	0	6	2	0
Sigma	5	7	3	2	0	0	0	0	0	0	0	1	1	1	2	0
Toxin	15	19	6	5	0	0	0	0	0	1	0	0	0	2	6	0
Transport	51	59	39	19	3	4	0	0	1	5	0	6	0	15	31	1
Transposon	119	99	54	62	6	4	6	4	11	36	6	13	7	6	50	2

Total	326	321	187	144	17	12	11	7	18	57	7	36	10	49	136	4

^a^Abe – *A. bestiarum*

^b^Aca – *A. caviae*

^c^Asa – *A. salmonicida *subsp. *achromogenes*

^d^Asm – *A. salmonicida *subsp. *masoucida*
